# Transcriptome Analysis of Mesenchymal Stem Cells from Multiple Myeloma Patients Reveals Downregulation of Genes Involved in Cell Cycle Progression, Immune Response, and Bone Metabolism

**DOI:** 10.1038/s41598-018-38314-8

**Published:** 2019-01-31

**Authors:** Rodrigo Carlini Fernando, Diego Robles Mazzotti, Hatylas Azevedo, Alex Freire Sandes, Edgar Gil Rizzatti, Mariana Bleker de Oliveira, Veruska Lia Fook Alves, Angela Isabel Pereira Eugênio, Fabrício de Carvalho, Maria Aparecida Dalboni, David Correa Martins, Gisele Wally Braga Colleoni

**Affiliations:** 1 Department of Experimental and Clinical Oncology, Discipline of Hematology and Hemotherapy, Federal University of São Paulo, UNIFESP, São Paulo, Brazil; 2 Center for Sleep and Circadian Neurobiology, University of Pennsylvania, Pennsylvania, USA; 3 Department of Pediatrics, Faculty of Medicine of the University of São Paulo, FMUSP, São Paulo, Brazil; 4 Hematology, Fleury, Medicine and Health, São Paulo, Brazil; 5 Departament of Post-Graduation in Medicine, University Nine of July, UNINOVE, São Paulo, Brazil; 6 Center of Mathematics, Computation and Congnition, Federal University of ABC, UFABC, Santo André, Brazil

## Abstract

A growing body of evidence suggests a key role of tumor microenvironment, especially for bone marrow mesenchymal stem cells (MSC), in the maintenance and progression of multiple myeloma (MM), through direct and indirect interactions with tumor plasma cells. Thus, this study aimed to investigate the gene expression and functional alterations of MSC from MM patients (MM-MSC) in comparison with their normal counterparts from normal donors (ND-MSC). Gene expression analysis (Affymetrix) was performed in MM-MSC and ND-MSC after *in vitro* expansion. To validate these findings, some genes were selected to be evaluated by quantitative real time PCR (RT-qPCR), and also functional *in vitro* analyses were performed. We demonstrated that MM-MSC have a distinct gene expression profile than ND-MSC, with 485 differentially expressed genes (DEG) - 280 upregulated and 205 downregulated. Bioinformatics analyses revealed that the main enriched functions among downregulated DEG were related to cell cycle progression, immune response activation and bone metabolism. Four genes were validated by qPCR - *ZNF521* and *SEMA3A*, which are involved in bone metabolism, and *HLA-DRA* and *CHIRL1*, which are implicated in the activation of immune response. Taken together, our results suggest that MM-MSC have constitutive abnormalities that remain present even in the absence of tumors cells. The alterations found in cell cycle progression, immune system activation, and osteoblastogenesis suggest, respectively, that MM-MSC are permanently dependent of tumor cells, might contribute to immune evasion and play an essential role in bone lesions frequently found in MM patients.

## Introduction

Multiple myeloma (MM) is a hematologic malignancy of plasma cells, characterized by the infiltration of tumor cells in the bone marrow (BM), production of monoclonal (M) protein, which can be detected in the blood and/or urine of patients, and lesions in target-tissues and organs, including hypercalcemia, renal failure, anemia, and bone lesions^1,2^. In the United States, MM represents, approximately, 1% of all cancer types and 10% of hematological malignancies^2^. The disease is often preceded by a pre-malignant condition known as monoclonal gammopathy of undetermined significance (MGUS)^3,4^, which is present in, approximately, 3% of the population over 50 years and represents a risk of progression to MM of 1% per year^5^. Smoldering MM is another pre-malignant condition that can be classified between MGUS and MM, and presents a higher risk of progression to MM (approximately, 10% per year)^6^.

The overall survival of MM patients has improved considerably in recent years, both for patients eligible for autologous hematopoietic stem cell transplantation as well as for those ineligibles for this therapeutic procedure^7,8^. However, these advances were not observed in all patients; for instance, among those classified as high-risk by molecular cytogenetic markers, the overall survival remains without significant advances^7^. In addition, most MM patients eventually relapse one or more times over the course of the disease, including those who have achieved a complete response, until the moment they might become refractory to all therapeutic arsenal available^7^.

The first breakthroughs in MM treatment were the introduction of melphalan in combination with prednisone, in the late 1960s^9^, and the introduction of high doses of chemotherapy followed by autologous hematopoietic stem cell transplantation for eligible patients, at the beginning of 1980s^10^. However, the greatest breakthrough occurred in 2000s, with the use of immunomodulatory agents, thalidomide^11^ and, subsequently, its analogues lenalidomide^12,13^ and pomalidomide^14^, as well as with the use of proteasome inhibitors, bortezomib^15^ and, more recently, carfilzomib^16^ and ixazomib^17^. Other therapeutic drugs have emerged, such as monoclonal antibodies daratumumab (anti-CD38)^18^ and elotuzumab (anti-SLAMF7)^19^, and histone deacetylase inhibitor panobinostat^20^. Besides, there are a large number of new drugs with great anti-MM potential that are being tested in preclinical and clinical studies, and, likely, the therapeutic arsenal for MM treatment is going to become even greater in the coming years.

Despite the great advance in MM treatment aforementioned, which has improved patients’ overall survival, MM remains an incurable disease and, therefore, more information about its pathogenesis is essential for the search for new therapeutic targets. Genetic and epigenetic alterations are often found in tumor plasma cells, and accumulate over the course of the disease^21^. However, several of these alterations are already found in plasma cells of patients with MGUS, and smoldering MM. Thus, it is intuitive to think that, although such alterations are necessary for MM development, they are not sufficient^21^. In this scenario, the role of the tumor microenvironment for MM pathogenesis emerges.

A growing body of evidence suggests that tumor microenvironment plays a key role in the maintenance and progression of various cancers, including solid tumors^22,23^ and hematological malignancies^24^. In MM, the essential role of BM tumor microenvironment is well established and several studies have shown that plasma cells strongly depend on it^25^. Tumor plasma cells interact, directly and indirectly, with the tumor microenvironment, which is composed by cellular and noncellular elements, promoting proliferation, migration, survival, and drug resistance^26–28^. Among cellular elements, mesenchymal stem cells (MSC) deserve great attention in MM pathogenesis^29^. MSC is an adult stem cell, that can be found in different organs or tissues, especially in BM^30^. Apparently, MSC from MM patients (MM-MSC) do not share the same genetic alterations present in MM cells, ruling out the hypothesis of a common progenitor^31^. However, an increasing but still limited number of studies have demonstrated that MM-MSC might have some important differences compared to MSC from normal donors (ND-MSC), comprising from differences in gene and protein expression, to functional alterations, including lower proliferation and osteoblastic differentiation capacity, impaired immunomodulatory properties, among others^31–35^.

Therefore, the aim of this study was to explore the differences between MM-MSC and their normal counterparts, through gene expression and functional analyses, in order to add new insights to MM pathogenesis that could contribute to the development of new therapeutic molecules capable of disrupting the interaction between MM-MSC and MM cells, making these tumors cells more sensitive to drugs and immune response action.

## Casuistic and Methods

### Ethical aspects

This study was approved by the Institutional Review Board  of the Federal University of São Paulo (CAAE: 34306314.6.0000.5505). BM samples were obtained after written informed consent of participants or legal representative, according to Helsinki Declaration and local regulations.

### Subjects and cell line

Nineteen patients, from both genders, newly diagnosed with MM and without any previous treatment for the disease, i.e., no chemotherapy, no corticosteroids, no immunomodulators, no proteasome inhibitors, or bisphosphonates, were successfully enrolled in this study and allocated in the case group. The clinical laboratory characteristics of MM patients at the diagnosis are shown in Table 1. Seven BM normal donors for allogeneic stem cell transplantation, not matched by age or gender, were also included in the study and allocated in the control group. As an additional control, the HS-5 human bone marrow normal stromal cell line (ATCC, Manassas, VA, USA) was also used in some experiments.

**Table 1:** Clinical and laboratorial characteristics of MM patients included in the study at diagnosis (N = 19).

Patients’ characteristics	
Median age, years (range)	67(43–80)
Sex, n (%)	
Male	09 (47)
Female	10 (53)
M-protein type, n (%)	
IgG	08 (42)
IgA	07 (37)
Light chain	04 (21)
D&S^a^ stage, n (%)	
I	01 (5)
II	02 (11)
III	16 (84)
ISS^b^ stage, n (%)	
1	04 (21)
2	04 (21)
3	08 (42)
NA^c^	03 (16)

^a^D&S = Durie & Salmon. ^b^ISS = International Staging System. ^c^NA = Not Available.

### Isolation, expansion and characterization of MSC

BM samples from normal donors (n = 7) and newly diagnosed MM patients (n = 19) were harvested from patients’ iliac crest. Then, BM mononuclear cells were isolated using Ficoll-Paque PLUS (GE Healthcare, Little Chalfont, Bucks, GBR), according to the manufacturer’s instructions. Finally, MSC were sorted by Magnetic-Activated Cell Sorting (MACS) methodology, using CD105^+^ as a positive marker (Miltenyi Biotec, Bergisch Gladbach, DEU). MSC expansion was performed on αMEM with GlutaMAX and nucleoside medium^36^, supplemented with penicillin (100 U/mL)/streptomycin (100 μg/mL), fungizone (2.5 μg/mL) (All Gibco, Carlsbad, CA, USA) and 10% fetal bovine serum (FBS) (Vitrocell, Campinas, SP, BRA). MSC were incubated at 37 °C, 5% CO_2_ and high humidity. During passage zero, the cells were fed twice a week, replacing only 50% of culture medium volume, until the cells reached 80% confluence or up to 21 days. Then, MSC were detached from the culture plastic with the aid of 0.25% trypsin-EDTA reagent (Gibco, Carlsbad, CA, USA), counted by tripan blue exclusion method and seeded again. From the first passage onwards, 80% of the culture medium volume was replaced at the same conditions. After expansion, MM-MSC, ND-MSC and HS-5 cell line were immunophenotyped in the BD FACSCanto II flow cytometer (Becton, Dickinson and Company, Franklin Lakes, NJ, USA). Positive and negative markers were chosen based on the International Society for Cell Therapy Criteria^37^, using the following monoclonal antibodies: anti-CD105 PE, anti-CD90 FITC, and anti-CD73 PE-Cy 7, as positive markers, and anti-CD45 PO, anti-CD34 PerCP-Cy 5.5, anti-CD14 APC-H7, and anti-HLA-DR PB, as negative markers. Data were acquired in the software FACSDIVA, version 8.0.1 (Becton, Dickinson and Company, Franklin Lakes, NJ, USA), and analyzed in the software Infinicyt, version 1.7  (Cytognos S. L., Salamanca, ESP).

The osteoblastic differentiation of MM-MSC (n = 4) and ND-MSC (n = 4) was performed in technical duplicates in 12-well microplates, using StemPro Osteogenesis Differentiation kit (Gibco, Carlsbad, CA, USA) and following the manufacturer’s instructions. Cells were fed twice a week, and on days 7, 14 and 21, the differentiation medium was removed and frozen at −80 °C for further analysis. In order to confirm the cell differentiation by a quantitative methodology, osteocalcin measurement was performed on the differentiation media collected from the cases and controls on days 7, 14 and 21, using the Human Osteocalcin Quantikine ELISA kit (R&D Systems, Minneapolis, MN, USA), according to the manufacturer’s instructions.

### Microarray hybridization and data acquisition

For gene expression analysis, RNA samples were extracted from MM-MSC (n = 4) and ND-MSC (n = 4) using RNeasy Mini kit (Qiagen, Valencia, CA, USA). For each sample, three independent RNA extractions were performed. RNA quantification and purity analysis were performed on the NanoDrop® ND-8000 UV spectrophotometer (NanoDrop Technologies, Wilmington, DE, USA). In addition, RNA integrity was verified by 1% agarose gel electrophoresis, stained with ethidium bromide.

The microarray platform used was GeneChip® Human Exon 1.0 ST Array (Affymetrix, Santa Clara, CA, USA). On this platform, there are about 1.4 million probe sets, being approximately 4 probes per exon and 40 probes per gene. Microarray images were obtained using GeneChip Scanner 3000 7 G, and data were quantified using Affymetrix GeneChip Command Console® Software (both Affymetrix, Santa Clara, CA, USA), generating CEL files containing the raw data.

### Microarray data preprocessing and analysis

Microarray raw data preprocessing and identification of differentially expressed genes (DEG) were performed using the AltAnalyze software www.altanalyze.org^38^. Once the analysis parameters were set, the raw data, in CEL format, was preprocessed by the Robust Multi-array Analysis (RMA) method^39^, which includes background correction, quantile normalization and summarization of the probes into specific probe sets. The probe sets were defined as differentially expressed between the groups when the p-values, corrected by the False Discovery Rate (FDR) method^40^, were less than 0.05 and the fold-changes (difference in expression in the case group *versus* control group) were greater than 1.5, in module. Differentially expressed probe sets were annotated for the purpose of identifying which genes they represent. To ensure that there was no great variability among within-condition samples, the coefficients of variation (CV), of the normalized gene expression values in log2, were calculated and, arbitrarily, the CV cut-off criteria less than 15% was established to consider a gene consistent. The microarray data, discussed in this article, have been deposited in NCBI’s Gene Expression Omnibus, and can be accessed through GEO Series accession number (ref GSE113736).

### Bioinformatics analyses workflow

After identification of DEG, we performed the bioinformatics analyses in order to extract relevant biological information among these genes.

#### Gene Co-Expression Network Analysis

Gene co-expression network construction and additional analyses were performed using Cytoscape 3.5.1 software^41^, and three of its plug-ins. First, the GeneMANIA plug-in^42^ was used to generate the network, through the prediction of interactions among DEG, based exclusively on data published in the literature concerning co-expression. Then, another plug-in, CentiScaPe^43^ was used to calculate centrality measures of the genes (nodes) belonging to the constructed network. In our study, the calculated centrality measures were degree and betweenness, which represent, respectively, the number of connections of a node, i.e., the number of interactions of a gene with other genes in the network, and the number of shortest paths that pass through a node to connect other pairs of nodes. Lastly, GLay plug-in^44^ was used to find modules, also known as communities or clusters, which means groups of highly interconnected genes in the network.

#### Identification of high-hubs, hubs and bottlenecks

The calculated degree and betweenness values were used to construct a scatter plot, using GraphPad Prism 7.0 statistical software (GraphPad Software, San Diego, CA, USA). The scatter plot allows categorization of nodes in high hubs, hubs, and bottlenecks, as previously described by Azevedo *et al*.^45^. In summary, by dividing the plot into quadrants, the genes located in the upper right quadrant represent the high hubs (high degree and betweenness values), whereas the genes located in the lower right quadrant represent the hubs (genes with high degree and low betweenness values), and, finally, genes located in the upper left quadrant represent bottlenecks (genes with high betweenness and low degree values). The most relevant functions of the nodes with the highest degrees and/or betweenness values were manually searched using the GeneCards database www.genecards.org.

#### Functional Enrichment Analysis

Overrepresented biological functions and pathways from GO^46,47^ and KEGG^48–51^ databases, respectively, were searched in the subnetworks found, with the aid of Enrichr software amp.pharm.mssm.edu/Enrichr/^52,53^ and DAVID, version 6.8, software https://david.ncifcrf.gov/^54^. P-value, adjusted for multiple comparisons by the FDR method^40^, less than 0.05 was used as cut-off criteria to consider a category as significantly enriched.

### Real-time RT-qPCR validation

In order to validate microarray results, total RNA extraction from MM-MSC (n = 13), ND-MSC (n = 5), and HS-5 cell line was performed using the RNA RNeasy Mini kit (Qiagen, Valencia, CA, USA), according to the manufacturer’s instructions. Then, cDNA was synthesized from 1.5 µg of total RNA, using SuperScript III and Oligo(dT) (both from Invitrogen, Carslbad, CA, USA). Real time RT-qPCR was carried out using the 7500 Real Time PCR System®, and the TaqMan Gene Expression Assays (both from Applied Biosystems, Foster City, CA, USA). All samples were evaluated in technical triplicates, and the Ct values for the endogenous control (*GAPDH*) and for the target genes (based on the bioinformatics analyses and their fold-change values), were determined during the log phase of the reaction. HS-5 cell line was used as a calibrator. For data analysis, the comparative ΔΔCt method was used^55^, where ΔCt = Ct_target gene_ − Ct_GAPDH_ and ΔΔCt = ΔCt_cases or controls_ − ΔCt_calibrator._ The candidate genes were considered differentially expressed in MM-MSC when their expression levels showed at least a 2-fold increase or decrease in comparison to ND-MSC.

### Telomere length measurement

Genomic DNA were extracted from MM-MSC (n = 19), ND-MSC (n = 7) and HS-5 cell line, using the Qiamp DNA Mini kit (Qiagen, Valencia, CA, USA), according to the manufacturer’s instructions. After extraction, DNA quantification and purity analysis were performed in the DS-10 spectrophotometer (DeNovix, Wilmington, DE, USA). Then, all DNA samples were diluted to a final concentration of 50 ηg/μL. MSC telomere length was determined by multiplex real time qPCR, as previously described by Cawthon^56,57^, with minor modifications. In summary, this methodology consists in determining the relative ratio (T/S) between the telomere region copy number (T) and a single copy gene (S), using a relative standard curve. In our study, we chose the *ALB* gene as the single copy gene. T/S ratio for each sample is proportional to the mean telomere length. All experiments were performed in triplicate and our CV inter-assay was around 13.04%.

### Cell cycle analysis

MM-MSC and ND-MSC frequencies distribution among cell cycle phases were evaluated in the BD FACSCanto II flow cytometer, using propidium iodide reagent (both Becton, Dickinson and Company, Franklin Lakes, NJ, USA). The results were analyzed using ModFit LT software (Verity Software House, Topsham, ME, USA).

### Statistical analyses

All statistical analyses were performed on IBM SPSS Statistics 20.0 software (IBM Corporation, Armonk, NY, USA), adopting α = 5% significance level. All graphs were plotted in GraphPad Prism 7 software (GraphPad Software, San Diego, CA, USA) and the results are shown as mean and standard deviation (SD). In order to evaluate the group effect (MM-MSC *versus* ND-MSC) over time (7, 14 and 21 days) on the measurements of the continuous variable osteocalcin, we used the Generalized Estimating Equation (GEE) with gamma distribution. Mann-Whitney U test was used to perform comparison among groups regarding relative gene expression by RT-qPCR. Additionally, to evaluate group effect on the continuous dependent variable mean telomere length (T/S), we used the independent t-test, as the probabilistic distribution of this variable was considered normal (p = 0.01, Kolmogorov-Smirnov test). We also assumed the homogeneous variance distribution between groups, since Levene’s test showed no significant difference between group variances (F = 0.053 and p = 0.819). Lastly, to investigate the existence of an association between the group (MM-MSC *versus* ND-MSC) and the relative frequency of cells in the different cell cycle phases (G0/G1, S and G2/M), we performed the Fisher’s exact two-tailed test, since some expected frequencies were less than five. Principal component (PCA) and t-distributed stochastic neighbor embedding (t-SNE) analyses were implemented in the R software in order to perform dimensionality reduction and assess how the samples group to each other.

## Results

### MSC phenotype and osteoblastic differentiation potential

**Figure 1:**
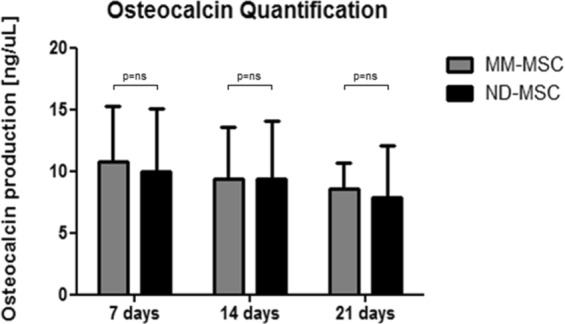
Comparison of osteocalcin production between MM-MSC (n = 4) and ND-MSC (n = 4), quantified by ELISA, on days 7, 14 and 21 of culture. The experiments were performed in technical duplicates and the results are presented as mean and standard deviation (SD). To evaluate the effect of the group over time on the osteocalcin measurements produced by MM-MSC and ND-MSC, the GEE method with gamma distribution was used. NS = Not Significant; MM-MSC = Multiple Myeloma-Mesenchymal Stem Cells; ND-MSC = Normal Donor-Mesenchymal Stem Cells.

MM-MSC and ND-MSC expressed CD105, CD90, and CD73 (positive markers), and did not express the negative markers CD45, CD34, CD14, and HLA-DR (data not shown). After *in vitro* induction for osteoblastic differentiation, it was possible to detect osteocalcin protein in the cell culture supernatant of MM-MSC (n = 4) and ND-MSC (n = 4) in the three moments evaluated (7, 14 and 21 days). The GEE statistical test with gamma distribution showed no statistically significant difference between osteocalcin measurements over time, synthesized *in vitro* by MM-MSC and ND-MSC (Fig. 1).

### Distinct gene expression profiling between MM-MSC and ND-MSC

After microarray data pre-processing and establishment of cut-off criteria (adjusted p-value < 0.05 and fold-change >1.5), we found 485 DEG between MM-MSC and ND-MSC, including 280 upregulated and 205 downregulated genes. About 50% of them correspond to protein-coding genes, while the other half comprised genes encoding for long non-coding RNAs, small nuclear RNAs, and small nucleolar RNAs. Arbitrarily, we considered that genes with the CV values less than 15%, i.e., with low variability within-condition, were consistent. Only 30 genes in the MM-MSC group and 28 genes in the ND-MSC were considered not consistent out of the 485 DEG, with an overlap of 12 non-consistent genes between the groups. The majority of the non-consistent genes were non-protein coding genes.

PCA and t-SNE analyses were performed to assess how the samples grouped to each other. Interestingly, both analyses were able to separate the samples into the studied groups, showing a good degree of similarity between the samples belonging to the same group (Figs S1 and S2**)**. The first two PCA components (PC1 and PC2) were able to explain 23.4% and 11.9% of the data, respectively (Fig. S3).

### Network and functional enrichment analyses reveal the functional gene signature of MM-MSC

The 485 DEG were used to build a gene co-expression network, where genes are represented as nodes, and their relationship, in this case co-expression, is represented as edges connecting the genes. The Cytoscape plug-in GeneMANIA was used to construct the network, using only interactions from the co-expression category. After filtering out the genes that were not connected in the network, we observed 195 genes (nodes) and 1515 interactions (edges) in the final network, with 31 genes upregulated, and 164 downregulated.

**Figure 2:**
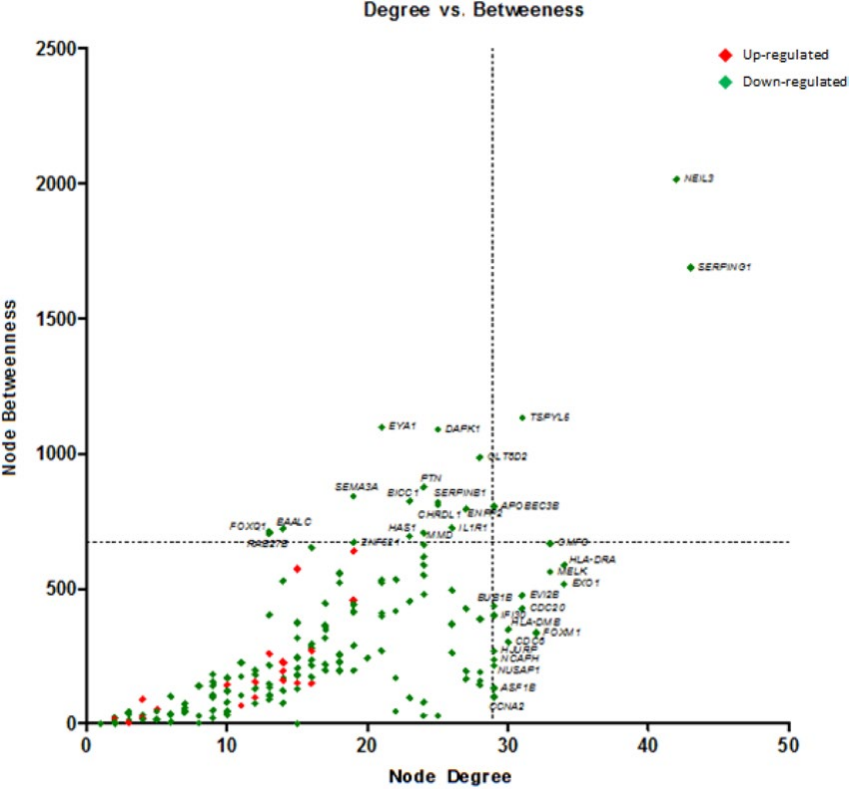
Scatter plot with degree and betweenness values for all nodes of gene co-expression network. The official gene symbol of the nodes with the highest values of degree (hubs) or betweenness (bottlenecks) are highlighted in the lower right and upper left quadrants, respectively. In addition, nodes with the highest degree and highest betweenness values (high hubs) are highlighted in the upper right quadrant. Red and green symbols represent, respectively, upregulated and downregulated genes.

**Figure 3:**
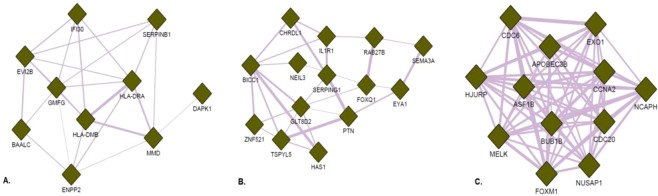
(**A**–**C**) Gene co-expression networks of the functional modules identified through the Cytoscape plug-in GLay, representing only the interactions between the hubs, bottlenecks and high hubs in each of the modules. Green color denotes gene dowregulation. All of the genes depicted above have decreased expression in MM-MSCs compared to ND-MSCs.

**Figure 4:**
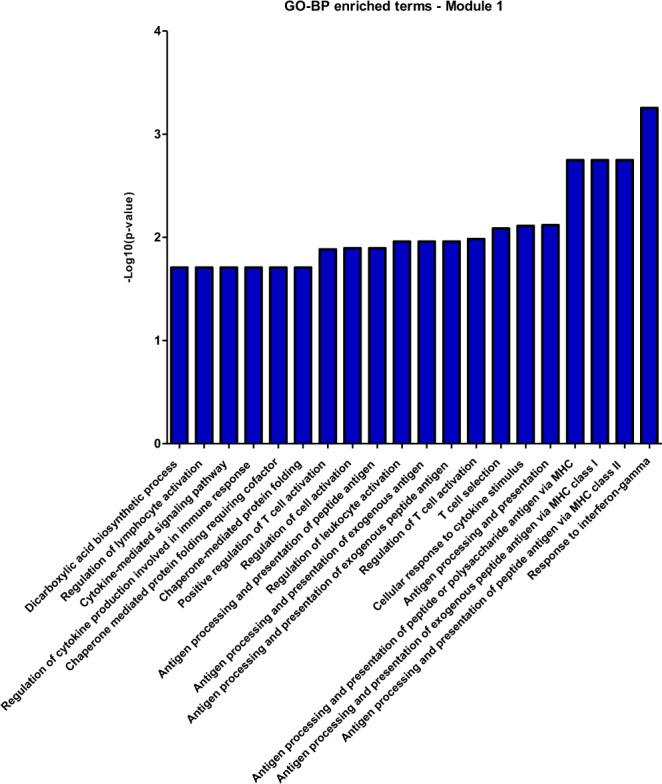
Co-expression network enrichment analysis showing overrepresented GO-BP terms for the network nodes from module 1. GO-BP = Gene Ontology-Biological Process.

**Figure 5:**
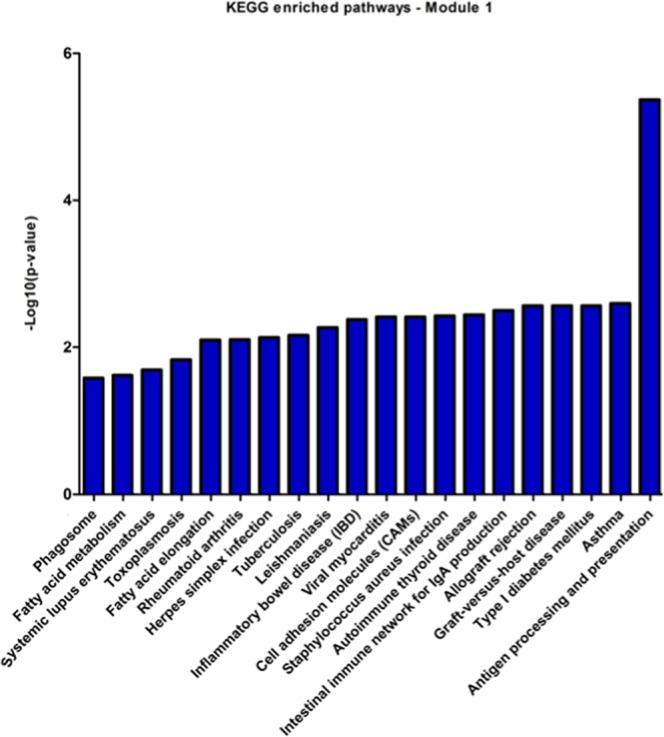
Co-expression network enrichment analysis showing overrepresented KEGG pathways for the network nodes from module 1. KEGG = Kyoto Encyclopedia of Genes and Genomes.

**Figure 6:**
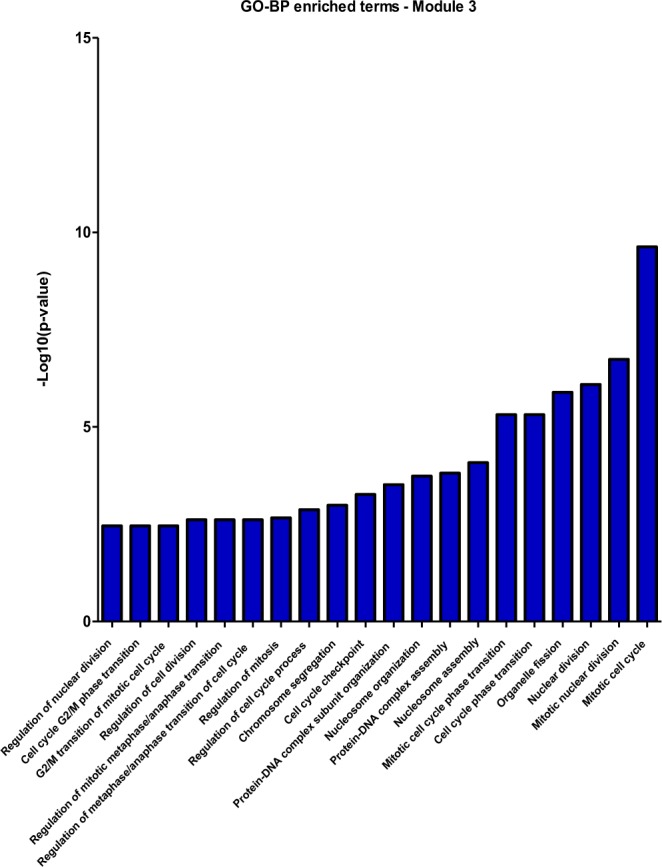
Co-expression network enrichment analysis showing overrepresented GO-BP terms for the network nodes from module 3. GO-BP = Gene Ontology-Biological Process.

**Figure 7:**
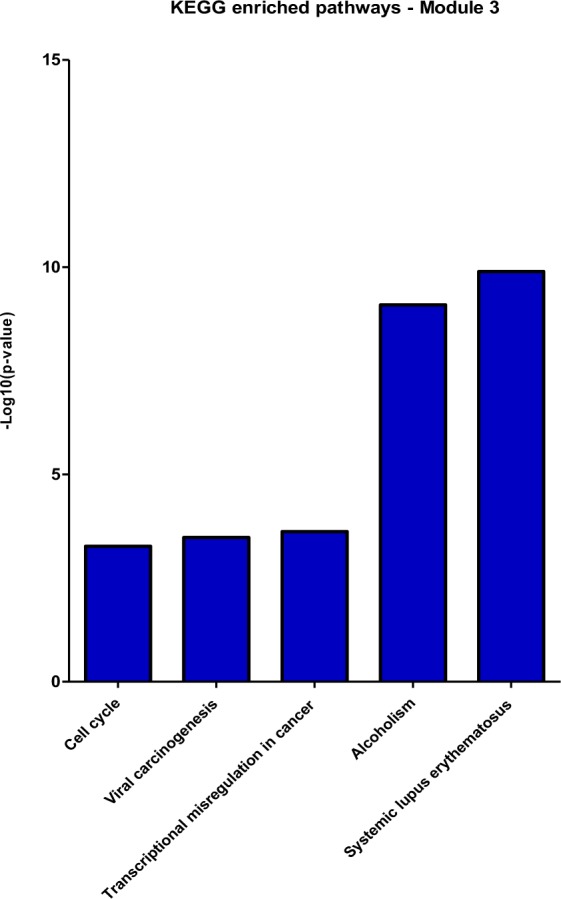
Co-expression network enrichment analysis showing overrepresented KEGG pathways for the network nodes from module 3. KEGG = Kyoto Encyclopedia of Genes and Genomes.

The degree and betweenness values of the 195 nodes were calculated, using the CentiScaPe plug-in, and a scatter plot was built to visualize the relationship between degree (x-axis) and betweenness (y-axis) for each node. The plot was divided into four quadrants by applying an arbitrary cut-off of degree ≥29 and betweenness ≥671.5 (Fig. 2). The 20 DEG with the highest degree (hubs) values and the 20 DEGs with the highest betweenness (bottlenecks) values were highlighted (total of 36 downregulated genes). Among the hubs, half of them participate directly or indirectly in cell cycle progression. Additionally, some other genes are involved in functions related to the immune response, such as antigen processing and presentation via MHC class II, and regulation of the complement system activation (Table 2). In parallel, bottleneck genes participate in cell cycle regulation or immune response. Besides, some genes are involved in osteoblastogenesis (*SEMA3A*, *BICC1*, *CHRLD1*, and *ZNF521*) and *HAS1* is involved in Waldenstrom’s macroglobulinemia (Table 3), a post-follicular B-cell lymphoproliferative disorder also associated with M-protein production (IgM). Finally, three genes with high degree and betweenness (high hubs) are involved in cell cycle progression, while one high-hub participates in the regulation of complement system activation (Table 4). We also performed the identification of functional modules within the gene co-expression network, i.e., the localization of groups of highly connected nodes inside the original network (Fig. 3). Enrichment analysis of the functional modules was performed using the Enrichr and the DAVID softwares. Since the results obtained from both softwares were very similar, i.e., there was a great overlap between the terms of GO and the biological pathways of KEGG significantly enriched, we decided to show only the results generated by Enrichr. Overrepresented GO biological processes and KEGG pathways are represented in Figs 4 and 5 for module 1, and in Figs 6 and 7 for module 3. We did not find any biological processes nor biological pathways significantly enriched in module 2. In module 1, enriched functions are mainly related to the immune response, T cell activation, antigen processing and presentation via MHC classes I and II, cytokine-mediated signaling pathways, and infectious and autoimmune diseases. In turn, enriched functions in module 3 are exclusively related to regulatory mechanisms of the cell cycle.

**Table 2:** The 16 DEG with the highest values of degree and low values of betweeness (hubs) in the gene co-expression network, and their most relevant functions.

Gene	Degree	Betweenness	Relevant function	Reference
*HLA-DRA*	34	589.1	Participates in immune response, through presenting peptides derived from extracellular proteins to immune cells	GeneCards
*EXO1*	34	515.9	Exonuclease which is involved in DNA mismatch repair, and is required for somatic hypermutation and class switch recombination of immunoglobulin genes	GeneCards and UniProtKB
*GMFG*	33	667.5	NA^a^	—
*MELK*	33	562.0	Serine/threonine-protein kinase which plays an important role in cell cycle regulation and carcinogenesis	UniProtKB
*FOXM1*	32	335.3	Transcriptional factor that regulates the expression of genes essential for cell cycle regulation	UniProtKB
*EVI2B*	31	475.0	NA	—
*CDC20*	31	426.8	Acts at multiple points during cell cycle, being required in nuclear movement prior to anaphase and chromosome segregation	Entrez Gene
*HLA-DMB*	30	347.0	Participates in immune response, by helping CLIP removal from the peptide binding site of MHC class II molecules	Entrez Gene
*CDC6*	30	302.5	Acts as a regulator of DNA replication and participates of checkpoints controls during cell cycle	Entrez Gene and UniProtKB
*BUB1B*	29	435.7	Kinase that plays a function as a cell cycle regulator, ensuring proper chromosome segregation before cell cycle progression	Entrez Gene
*IFI30*	29	401.4	Lysosomal thiol reductase involved in immune response, through MHC class II-restricted antigen processing	Entrez Gene
*HJURP*	29	269.5	Centromeric protein related with chromosome maintenance and cell cycle	GeneCards
*NCAPH*	29	236.6	Represents the regulatory subunit of the condensin complex, which is required for conversion of interphase chromatin into condensed chromosomes for mitosis progression	Entrez Gene and UniProtKB
*NUSAP1*	29	213.9	Promotes the organization of mitotic spindle microtubules around chromosomes	Entrez Gene
*ASF1B*	29	131.2	Histone chaperone that promotes histone deposition, exchange and removal during nucleosome assembly and disassembly	Entrez Gene
*CCNA2*	29	99.5	Involved in cell cycle control, promoting transition through G1/S and G2/M and it is associated with cellular senescence pathway	Entrez Gene, GeneCards and UniProtKB

Keywords are underlined. ^a^NA = Not Available.

**Table 3:** The 16 DEG with the highest values of betweeness and low values of degree (bottlenecks) in the gene co-expression network, and their most relevant functions.

Gene	Degree	Betweenness	Relevant function	Reference
*EYA1*	21	1098.0	Acts as protein phosphatase and as transcriptional coactivator, participating in DNA double-strand break repair	GeneCards and UniProtKB
*DAPK1*	25	1090.5	Serine/Threonine kinase that participates in cell survival, apoptosis, and autophagy	UniProtKB
*GLT8D2*	28	986.2	NA^a^	—
*PTN*	24	876.9	Secreted growth factor that plays essential roles in several pathways, including survival, cell migration, angiogenesis, and tumorigenesis	Entrez Gene
*SEMA3A*	19	842.6	Positive regulator of osteoblastogenesis	Hayashi *et al*.^80^
*BICC1*	23	824.2	Acts as a genetic determinant of osteoblastogenesis and bone mineral density	Mesner *et al*.^78^
*SERPINB1*	25	822.2	Proteinase inhibitor that participates of innate immune response, being a potent intracellular inhibitor of granzyme H and regulating the activity of neutrophil proteases	Entrez Gene and UniProtKB
*CHRDL1*	25	809.0	Antagonist of bone morphogenetic protein 4	Entrez Gene
*ENPP2*	27	796.0	Phopholipase which is responsible for catalyzing the production of lysophosphatidic acid in extracellular fluids, promoting cell proliferation and chemotaxis	Entrez Gene
*IL1R1*	26	725.5	Cytokine receptor, being an important mediator involved in immune response and inflammatory responses	Entrez Gene
*BAALC*	14	722.2	Indetified in patients with acute myeloid leukemia	Entrez Gene
*FOXQ1*	13	712.0	Involved in cell cycle regulation and tumorigenesis	Entrez Gene
*MMD*	24	707.0	Molecule expressed by *in vitro* differentiated macrophage	Entrez Gene
*RAB27B*	13	703.3	Belongs to the Rab protein family and plays a role in vesicular fusion and trafficking	Entrez Gene
*HAS1*	23	694.4	Enzyme that catalyzes the hyaluronan synthesis, a major component of most extracellular matrices. It is also associated with Waldenstrom macroglobulinemia	Entrez Gene, GeneCards, and UniProtKB
*ZNF521*	19	671.5	Zinc finger protein that promotes the maturation and function of mature osteoblasts	Hesse *et al*.^75^

Keywords are underlined. ^a^NA = Not Available.

**Table 4:** The 4 DEG with the highest values of degree and betweeness (high hubs) in the gene co-expression network, and their most relevant functions.

Gene	Degree	Betweenness	Relevant function	Reference
*NEIL3*	42	2016.0	Plays a role in cell cycle, by repairing telomere damage during phase S	Zhou *et al.*^81^
*SERPING1*	43	1687.6	Protein involved in the regulation of complement system activation	Entrez Gene
*TSPYL5*	31	1133.1	Involved in modulation of cell growth, through TP53/p53 inhibition	UniProtKB
*APOBEC3B*	29	805.4	Might participate in cell growth and/or cell cycle control	Entrez Gene

Keywords are underlined.

### Real-time RT-qPCR validation

**Figure 8:**
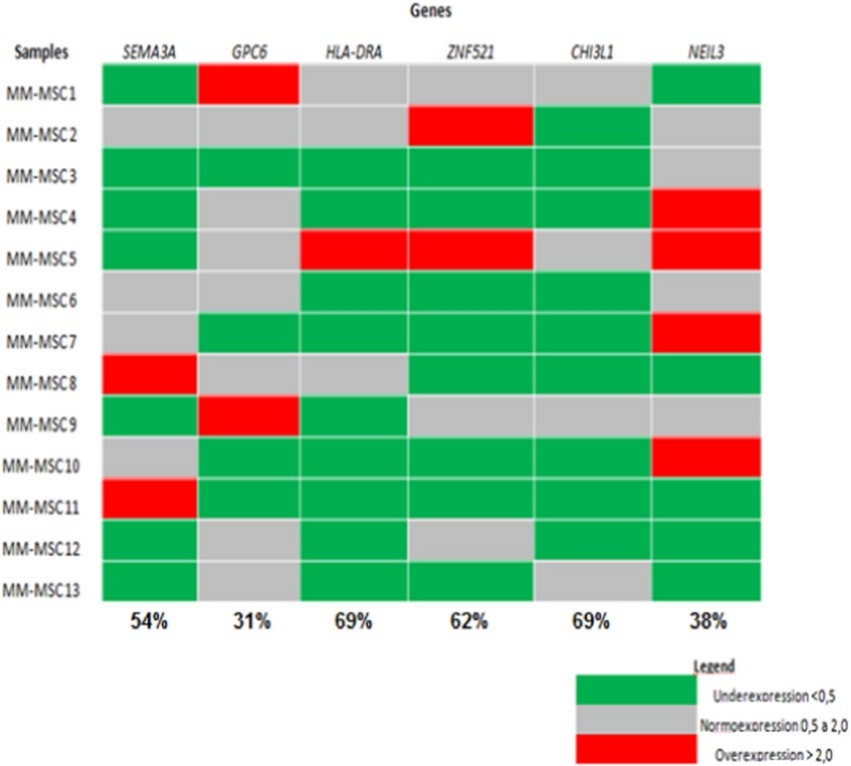
Heat-map showing the expression pattern of the genes *SEMA3A*, *GPC6*, *HLA-DRA*, *ZNF521*, *CHI3L1*, and *NEIL3*, evaluated by RT-qPCR. The candidate genes were considered differentially expressed in MM-MSC when their expression levels showed at least a 2-fold increase or decrease in comparison to normal cells. MM-MSC = Multiple Myeloma Mesenchymal Stem Cells.

**Figure 9:**
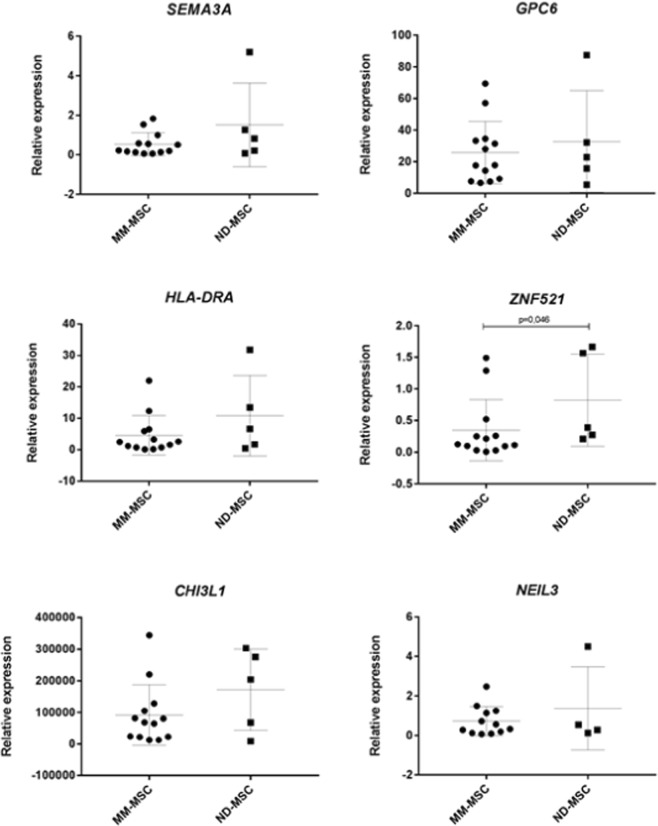
Expression of *SEMA3A*, *GPC6*, *HLA-DRA*, *ZNF521*, *CHI3L1*, and *NEIL3*, evaluated by RT-qPCR, of MM-MSC (n = 13) in comparison with ND-MSC (n = 5). The experiments were performed in technical triplicates and the results are presented as mean and standard deviation (SD). *GAPDH* gene was used as endogenous control and the HS-5 cell line as a calibrator. Mann-Whitney U test was used to perform comparison among groups regarding relative gene expression. NS = Not Significant; MM-MSC = Multiple Myeloma-Mesenchymal Stem Cells; ND-MSC = Normal Donor-Mesenchymal Stem Cells.

The microarray data were validated at the mRNA level by RT-qPCR for *SEMA3A*, *ZNF521*, *HLA-DRA*, *CHI3L1*, *NEIL3*, and *GPC6*. The genes *HLA-DRA*, *CHI3L1*, *and ZNF521* were downregulated in 69%, 69% and 62% of MM-MSC, whereas genes *SEMA3A*, *NEIL3 and GPC6* were downregulated in 54%, 38%, and 31% of MM-MSC, respectively (Fig. 8). Although all genes were downregulated in tumor samples when compared with normal controls, we only found a statistically significant difference for the *ZNF521* gene (p = 0.046) (Fig. 9).

### Multiple myeloma effects on MM-MSC telomere length

**Figure 10:**
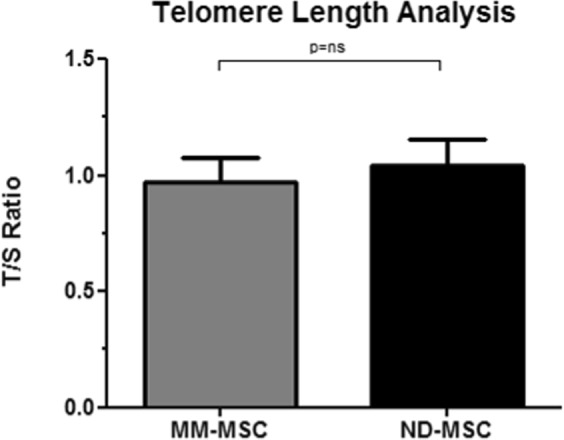
Mean telomere length of MM-MSC (n = 19) in comparison with ND-MSC (n = 7), expressed by T/S ratios. The experiments were performed in technical triplicates and the results are presented as mean and standard deviation (SD). To determine the effect of group on MSC telomere length, the independent t-test was used. NS = Not Significant; MM-MSC = Multiple Myeloma-Mesenchymal Stem Cells; ND-MSC = Normal Donor-Mesenchymal Stem Cells.

Telomere length comparison between MM-MSC and ND-MSC are showed in Fig. 10. As expected, MM-MSC presented lower telomeric length (mean = 0.97, SD = 0.11) than ND-MSC (mean = 1.04, SD = 0.11). However, the independent t test for the two samples showed that the difference found was not statistically significant (t_(24)_ = 1.578, p = 0.128).

### Cell cycle analysis showed no difference between MM-MSC and ND-MSC

**Figure 11:**
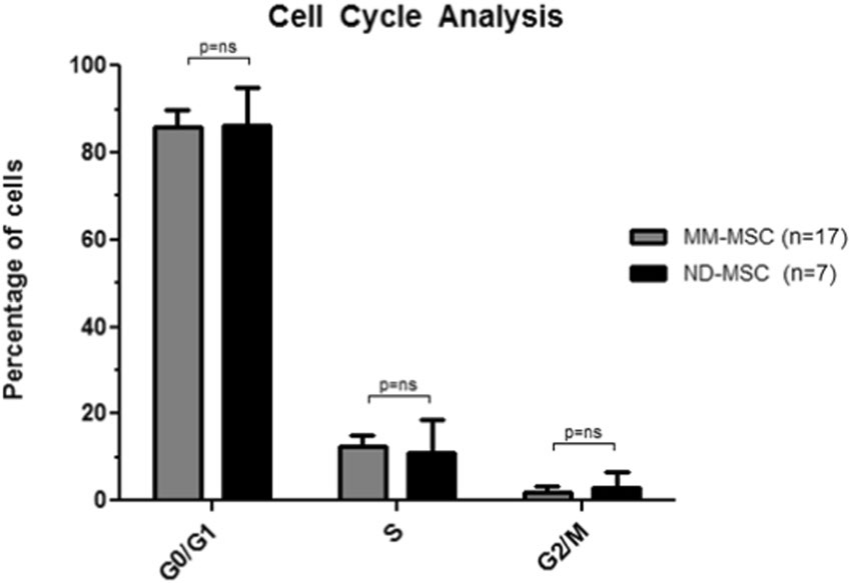
Cell cycle analysis of MM-MSC (n = 17) in comparison with ND-MSC (n = 7), expressed by cell percentages over G0/G1, S and G2/M phases. The results are presented as mean and standard deviation (SD). To evaluate the association of the group and the MSC frequencies over cell cycle phases, the Fisher’s exact two-tailed test was used. NS = Not Significant; MM-MSC = Multiple Myeloma-Mesenchymal Stem Cells; ND-MSC = Normal Donor-Mesenchymal Stem Cells.

The majority of MSC from cases and controls were in the quiescent phase (G0/G1 phase, MM-MSC: mean = 86.05%, SD = 8.97%, ND-MSC: mean = 85.82%, SD = 4.01%), with a few cells in S phase (MM-MSC: mean = 10.93%, SD = 7.74, ND-MSC: mean = 12.32%, SD = 2.87%), and in the G2/M phase (MM-MSC: mean = 3.02%, SD = 3.61%, ND-MSC: mean = 1.86%, SD = 1.33%) (Fig. 11). Fisher’s exact two-tailed test showed no statistically significant difference between the MM-MSC and ND-MSC percentages in the G0/G1, S and G2/M cell cycle phases (p = 1.00).

## Discussion

Gene expression profiling analysis of MM-MSCs compared to ND-MSCs revealed 485 DEG, being 280 upregulated and 205 downregulated. When we built the co-expression network, there was a reversal and the downregulated genes became more represented in the network than the upregulated (164 *versus* 31), which was expected, since among the 280 upregulated genes, only 49 were protein-coding genes, whereas, of the 205 downregulated ones, 170 were protein-coding genes. Further exploration of DEG with different bioinformatics tools, showed that the most relevant enriched pathways and functions were among the downregulated genes, especially for those involved in cell cycle progression, immune response activation, and osteoblastic function and maturation. We have validated part of these findings through real-time quantitative PCR methodology, and additional *in vitro* functional assays. Six downregulated target genes, whose functions were mainly related to cell cycle progression, immune response activation, and osteoblastic function and maturation, were selected for validation at the mRNA level. Of the six target genes selected, a statistically significant difference was detected only for the *ZNF521* (p = 0.048), which was downregulated in 62% of the MM-MSC evaluated samples, validating the microarray findings. In line with these results, a tendency to down expression was also observed for the *HLA-DRA* and *CHI3L1* genes (both 69%), and for *SEMA3A* gene (54%). Regarding the functional *in vitro* assays, we chose to validate the cell cycle progression, since this function has already been reported by other authors as being impaired in MM-MSC, leading these cells to enter into an early cellular senescence process when compared to ND-MSC. To perform the functional validation, we analyzed the cell cycle phases distribution, and the telomeric length quantification, through, respectively, flow cytometry, and multiplex real-time quantitative PCR methodologies. Regarding the cell cycle distribution, the majority of MM-MSC and ND-MSC were quiescent G0/G1 (mean = 86.05%, SD = 8.97%). The mean telomeric length was lower in MM-MSC in comparison with the ND-MSC (mean = 0.97, SD = 0.11 *versus* mean = 1.04, SD = 0.11). However, the difference found was not statistically significant, suggesting that the alterations in mRNA level related to the cell cycle are not related to a shortening of telomeres, or the casuistic was insufficient to detect this difference due to the biological variability among individuals.

Our results suggest that MM-MSCs have a distinct gene expression profiling in comparison with ND-MSCs. Interestingly, these cells showed hundreds of DEG even after *in vitro* expansion in the absence of tumor cells, confirming previous findings^32–34,58^ (Table 5). However, as discussed by André *et al*.^33^, the variability of the results found in these studies is relatively high, possibly due to several factors, including but not limited to: methodological differences prior to microarray execution - from the local and the form of MSC isolation, as well as its method of cultivation - up to differences in the microarray platform chosen, as well as the statistical and bioinformatics approaches used for the pre-processing of the raw data and identification of the DEG. However, despite the differences, it is still possible to make relevant comparisons among these studies, and to raise quite pertinent hypotheses.

**Table 5:** Microarray studies addressing gene expression profiling of MM-MSC compared to ND-MSC.

Casuistic	Comparison design	*Microarray* *plataform*	Number of DEG^a^/*Probesets*	Reference
ND^b^ = 7 MM^c^ = 6	MM-MSC^d^ vs. ND-MSC^e^	GeneChip Human Genome U133 Plus 2.0 Array	183	Corre *et al*.^32^
ND = 7 MM = 16	MM-MSC vs. ND-MSC	GeneChip Human Genome U133A Array	79	Todoerti *et al*.^58^
ND = 3 MM = 4	MM-MSC vs. ND-MSC	GeneChip Human Genome U133 Plus 2.0 Array	646	André *et al*.^33^
ND = 8 MM = 14	(I) [(coMM-MSC¹ vs. MM-MSC - MM-MSC vs. ND-MSC) ∩ (coND-MSC² vs. ND-MSC - MM-MSC vs. ND-MSC)] (II) coMM-MSC vs. MM-MSC exclusive	GeneChip Human Genome U133 Plus 2.0 Array	(I) 2583/ (II) 2553	Garcia-Gomez *et al*.^34^
ND = 4 MM = 4	MM-MSC vs. ND-MSC	GeneChip Human Exon 1.0 ST Array	485	Fernando *et al*.(present study)

^a^DEG = Differentially Expressed Genes; ^b^ND = Normal Donor; ^c^MM = Multiple Myeloma; ^d^MM-MSC = Multiple Myeloma-Mesenchymal Stem Cells; ^e^ND-MSC = Normal Donor-Mesenchymal Stem Cells.

The first work to evaluate the overall gene expression profiling of MM-MSC, in comparison with ND-MSC, was published in 2007 by Corre *et al*.^32^. Among the 183 DEG/Probesets found, 59 were classified by the authors as belonging to the category of tumor microenvironment, comprising functions such as cellular communication, receptor signaling molecules, extracellular matrix, and secretory molecules. Additionally, they highlighted 40 genes (20 upregulated and 20 downregulated) as being essential for MM, of which four were also found in our study, one gene upregulated - *ANGPTL4* - and three with diminished expression - *NPR3*, *TNFRSF19*, and *FBLN1*. The gene *ANGPTL4* was also found downregulated in MM-MSC in three previous independent studies^32–34^. More recent publications have demonstrated its multiple roles in osteolytic lesions^59^, and MM bone disease^60^, for example, through the promotion of osteoclast-mediated bone resorption, cartilage degradation, and angiogenesis. The *NPR3* and *FBLN1* genes, among other functions, participate in bone formation^61,62^, i.e., the reduction of their expression can potentially contribute to the development of osteolytic lesions frequently found in patients with MM. Finally, the *TNFRSF19* gene, a member of the TNF receptor superfamily, appears to mediate caspase-independent cell death (Gene database, NCBI).

In line with our results, André *et al*.^33^ also found enriched categories related to the cell cycle, such as, “M-phase”, “DNA replication”, “cell cycle regulation”, etc, among downregulated genes, like those results found by Wagner *et al*. (2008) in a study with ND-MSC in replicative senescence^63^. In our study, one of the functional modules detected through Cytoscape was composed mainly of genes involved in different pathways and biological processes related to cell cycle progression. In addition, André *et al*^33^. also demonstrated that MM-MSCs have a lower proliferative rate, higher cell size, β-galactosidase increased activity, retention of cells in the S phase of the cell cycle, and secrete a senescence-associated molecule profile. Thus, the authors hypothesized that MM-MSCs appear to become senescent earlier than ND-MSCs. It is important to highlight that in this study, MSCs were expanded *in vitro* in monoculture, i.e. in the absence of other cells including MM plasma cells. Berenstein *et al*.^64^ demonstrated that MM-MSC, when cultured in the absence of tumor cells, accumulate in the S-phase of the cell cycle, increase β-galactosidase activity, and increase the expression of microRNAs associated with senescence. Moreover, they also demonstrated that MM-MSC co-cultivation with a MM cell line (KMS12-PE) is able to reverse, at least partially, the senescence phenotype of MM-MSC^64^. Contributing to these findings, the study of Garcia-Gomez *et al*.^34^, which evaluated the gene expression profiling of MM-MSC expanded in monoculture and co-culture with the MM cell line MM.1 S, observed that, after co-cultivation, some genes related to cell cycle progression become upregulated.

With regard to the other functional module identified through Cytoscape after performing functional enrichment analysis, we observed that the majority of the enriched categories was related to different aspects of the immune response, including antigen processing and presentation via MHC classes I and II, T cells activation, and immune response triggered by inflammatory cytokines. All genes belonging to these categories were downregulated in MM-MSC, suggesting that these biological pathways and processes could figure as a possible mechanism of immune escape. The immune system of MM patients is highly impaired^65^. A study published by our group showed that in BM of MM patients there is an increased expression of Treg cell markers, *FOXP3* and *CTLA4* genes, suggesting their accumulation in the BM microenvironment of MM patients, and a possible mechanism of tumor evasion from the immune system^66^. Besides, other studies have showed that the decrease in the number of CD19+ B-cells, CD4+ and CD8+ T-cells in patients with MM is negatively correlated with survival in these individuals^67–69^. Moreover, several studies have demonstrated a decrease in the number and/or function of different types of CD4+ and CD8+ T-cells in patients with MM^70^. Another cell type affected in several types of cancer, including in MM, are dendritic cells, which mediate antigen-specific immune responses, through the antigen processing and presentation via MHC class II to T-cells^71,72^. Magalhães *et al*.^73^ demonstrated that patients who achieved long-term control of MM have several differences in the immune system compared to patients with active disease, ranging from increased cytotoxic T cells and NK cells, to decreasing numbers of Treg cells, thus favoring the restoration of the antitumor cytotoxic responses of the immune system. The MSCs, under normal conditions, play an important immunoregulatory role. In the context of multiple myeloma, although they are not the tumor cells themselves, growing evidence from the literature has shown that their immunoregulatory functions are altered. A recent study by Chen *et al*.^74^, demonstrated in animal model that MM-MSCs promote the proliferation of tumor plasma cells through the inhibition of T-cell mediated immune responses via the PD-1 and PD-L1 pathway. Therefore, although no functional experiments have been performed to assess the immune response in MM-MCS, based on literature evidence, genes related to different pathways of the immune system, including antigen processing and presentation, are expected to be altered in MM-MSC when compared to ND-MSC, as was observed in our study. However, the meaning of these alterations, as well as their role in the pathophysiology of the disease, still need to be elucidated.

Regarding bone formation and resorption, the *ZNF521* gene, which was downregulated in MM-MSCs, plays a key role in bone metabolism - this gene acts inhibiting the differentiation of osteoblast progenitors, through binding to *RUNX2* pro-differentiation transcription factor, and simultaneously promoting maturation and correct function of mature osteoblasts^75^. In agreement with these data, the *RUNX2* gene was also downregulated in MM-MSC (FC = −1.65, gene expression evaluated only by microarray), possibly contributing to the imbalance of the bone metabolism observed in patients with MM. In addition, the downregulation of *SEMA3A*, which was classified as a bottleneck in the co-expression network, may also contribute to bone metabolism imbalance, since it plays an important role in osteoblastogenesis, inhibiting osteoclastic differentiation and stimulating osteoblastic differentiation^76^. This gene also acts as an inhibitor of angiogenesis in endothelial cells, and a study reported that the loss of its inhibitory capacity may contribute to the transition from MGUS to the active form of MM^77^. Finally, the *BICC1* gene, also downregulated in the MM-MSC and classified as a bottleneck, is a genetic determinant of osteoblastogenesis and mineral bone density^78^.

The main limitation of our study is the small number of MM patients enrolled (n = 19). All of them were diagnosed in the same public hospital which receives most of MM new cases in stage III (84% in this study) and medical emergency, such as spinal cord compression, renal insufficiency, hypercalcemia or bone fractures, when immediate therapeutic interventions with corticosteroids and bisphosphonates are necessary, making patients ineligible for gene expression studies. Another limitation was that the controls were not age-matched to cases. In general, transplant normal donors were younger than MM patients. This situation could raise the hypothesis that the early senescence profile of MM-MSC, or the other differences detected through bioinformatics analysis, could be artificially created by the lack of age matching. Magalhães *et al*.^79^ conducted a meta-analysis of microarray studies that evaluated aging-related genes, and they identified 74 genes with higher levels of evidence. Of the 485 DEG in the MM-MSC compared to the ND-MSCs identified in this study, there was an overlap of only two genes within the 74 reported by Magalhães *et al*.^79^. The genes were *SERPING1* and *S100A6*, which are involved with the regulation of complement cascade and cell cycle progression and differentiation, respectively. Thus, the absence of age-matched cases and controls probably did not significantly affect our data. Additionally, MSC from both groups were expanded *in vitro*, which might introduce artifacts. However, unfortunately, MSC from normal donors and patients with MM are found in very low number in the BM. Therefore, in order to obtain the appropriate number of cells to perform experiments, these cells must be expanded *in vitro* previously. Finally, MM-MSCs were expanded *in vitro* in the absence of MM tumor cells. However, despite this limitation, the comparisons among different studies, including those that were carried out in co-culture, allow the researchers to generate quite interesting hypotheses, which can be tested through comparisons between the monoculture and the co-culture of the MM- MSC with or without MM cells.

In summary, our study demonstrated that MM-MSCs have a distinct gene expression profile when compared to the ND-MSCs, corroborating previous studies. The functional enrichment analysis of the gene co-expression network revealed that the main deregulated functions in MM-MSC are related to cell cycle progression, activation of the immune response, and to bone metabolism, which may contribute directly or indirectly to MM physiopathology. Due to the essential role of these cells in the maintenance and progression of MM, potential therapeutic targets and new drugs capable of disrupting the interactions between MM-MSC and MM cells are welcome.

## Supplementary information

Supplemental Figures
