# Supplementary material for: Transcriptome Analysis of Mesenchymal Stem Cells from Multiple Myeloma Patients Reveals Downregulation of Genes Involved in Cell Cycle Progression, Immune Response, and Bone Metabolism
Source: Sci Rep. 2019 Jan 31;9:1056. doi: 10.1038/s41598-018-38314-8 (PMC6355867; doi:10.1038/s41598-018-38314-8)
Supplement: Supplementary file 1 — Supplemental Figures [file 41598_2018_38314_MOESM1_ESM.docx]

Manuscript title: **TRANSCRIPTOME ANALYSIS OF MESENCHYMAL STEM CELLS FROM MULTIPLE MYELOMA PATIENTS REVEALS DOWNREGULATION OF GENES INVOLVED IN CELL CYCLE PROGRESSION, IMMUNE RESPONSE, AND BONE METABOLISM**

Authors: **Fernando RC, PhD, Mazzoti DR, PhD, Azevedo H, PhD, Sandes AF, MD, PhD, Rizzatti EG, MD, PhD, de Oliveira MB, PhD, Alves VLF, PhD, Eugênio AIP, PhD, de Carvalho F, PhD, Dalboni MA, PhD, Martis DC, PhD and Colleoni GWB, MD, PhD.**


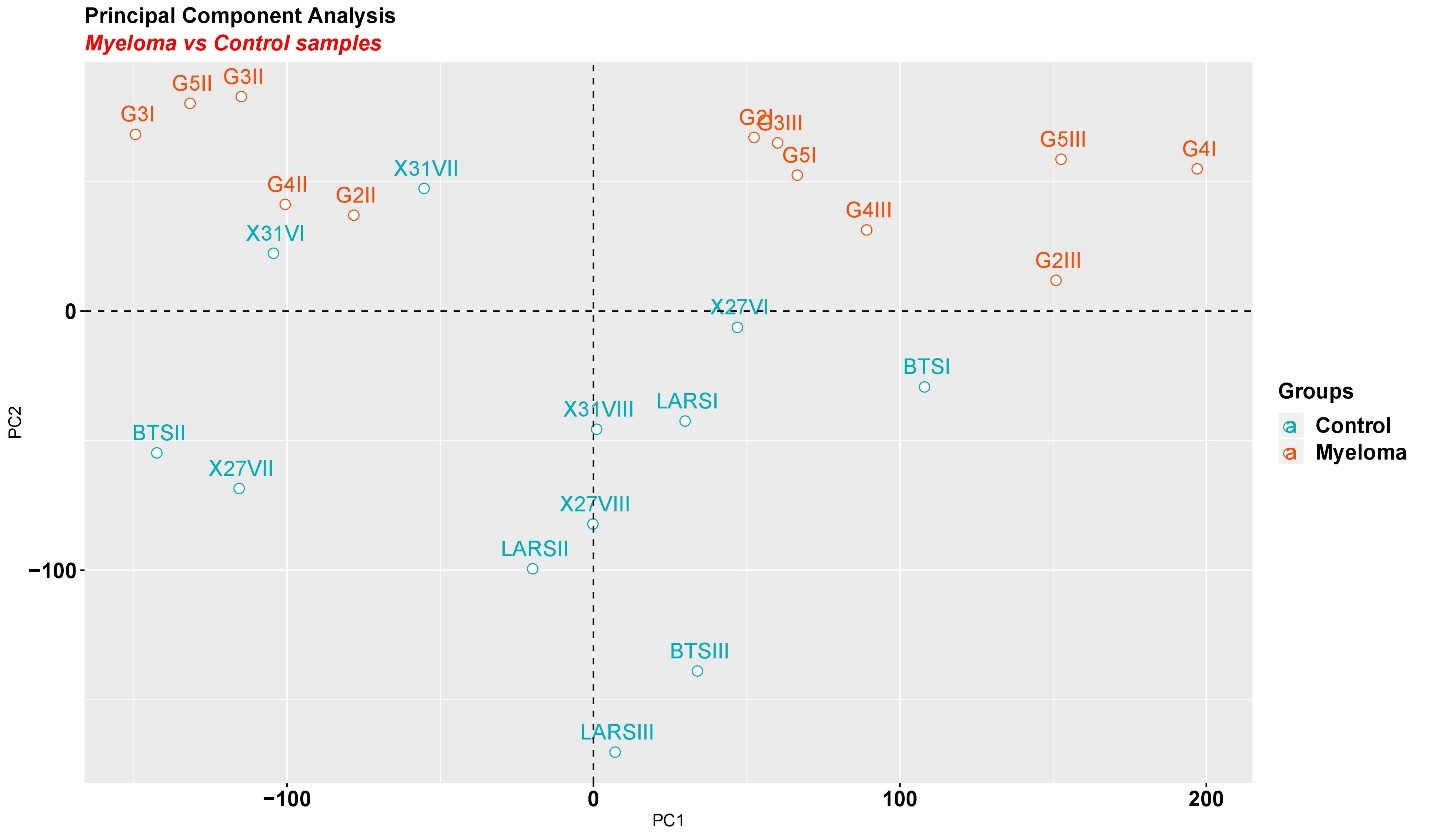


**Figure S1. Principal component analysis, in order to perform dimensionality reduction and to assess how the samples, MM-MSC and ND-MSC, group to each other.**


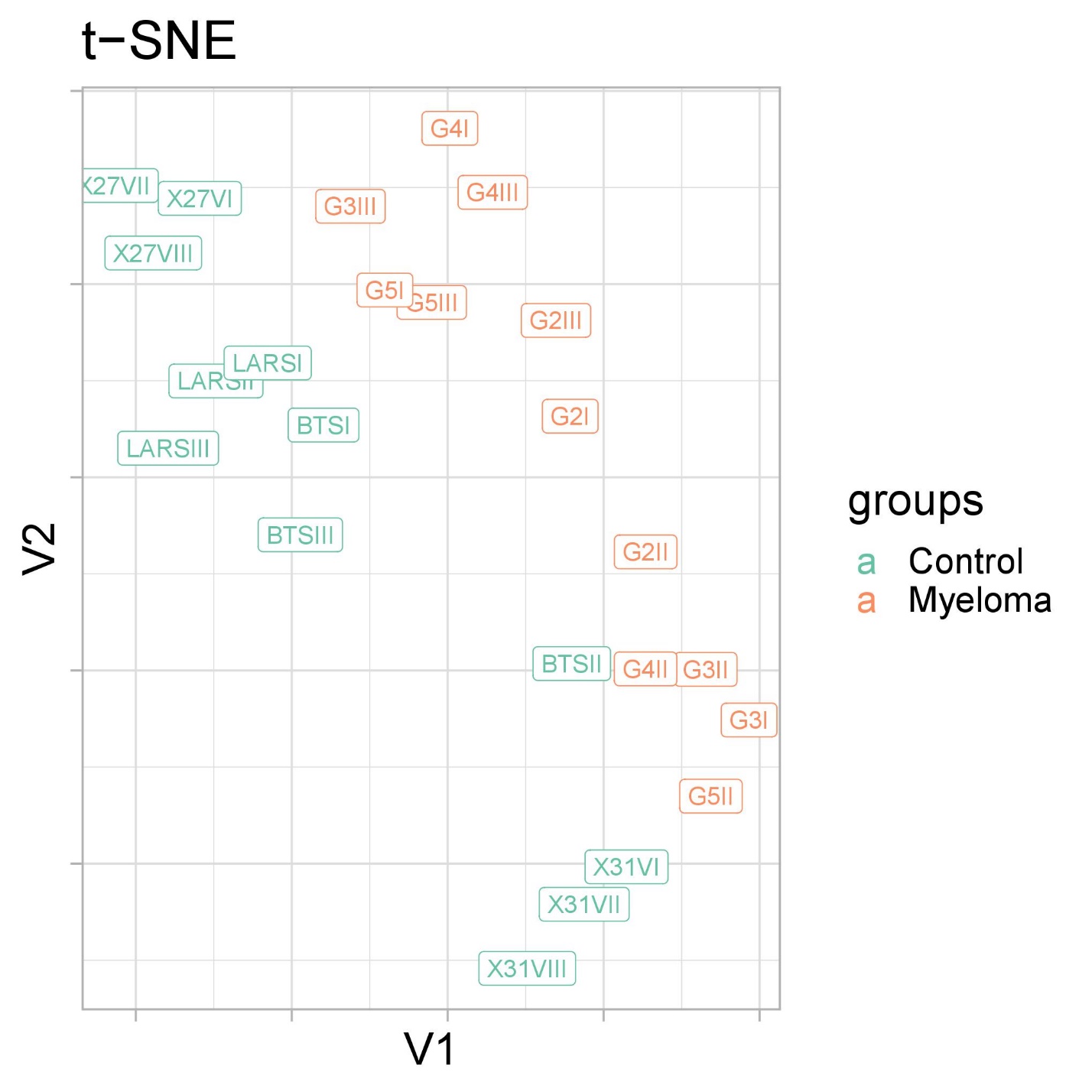


**Figure S2. T-distributed stochastic neighbor embedding analysis, in order to perform dimensionality reduction and to assess how the samples, MM-MSC and ND-MSC, group to each other.**


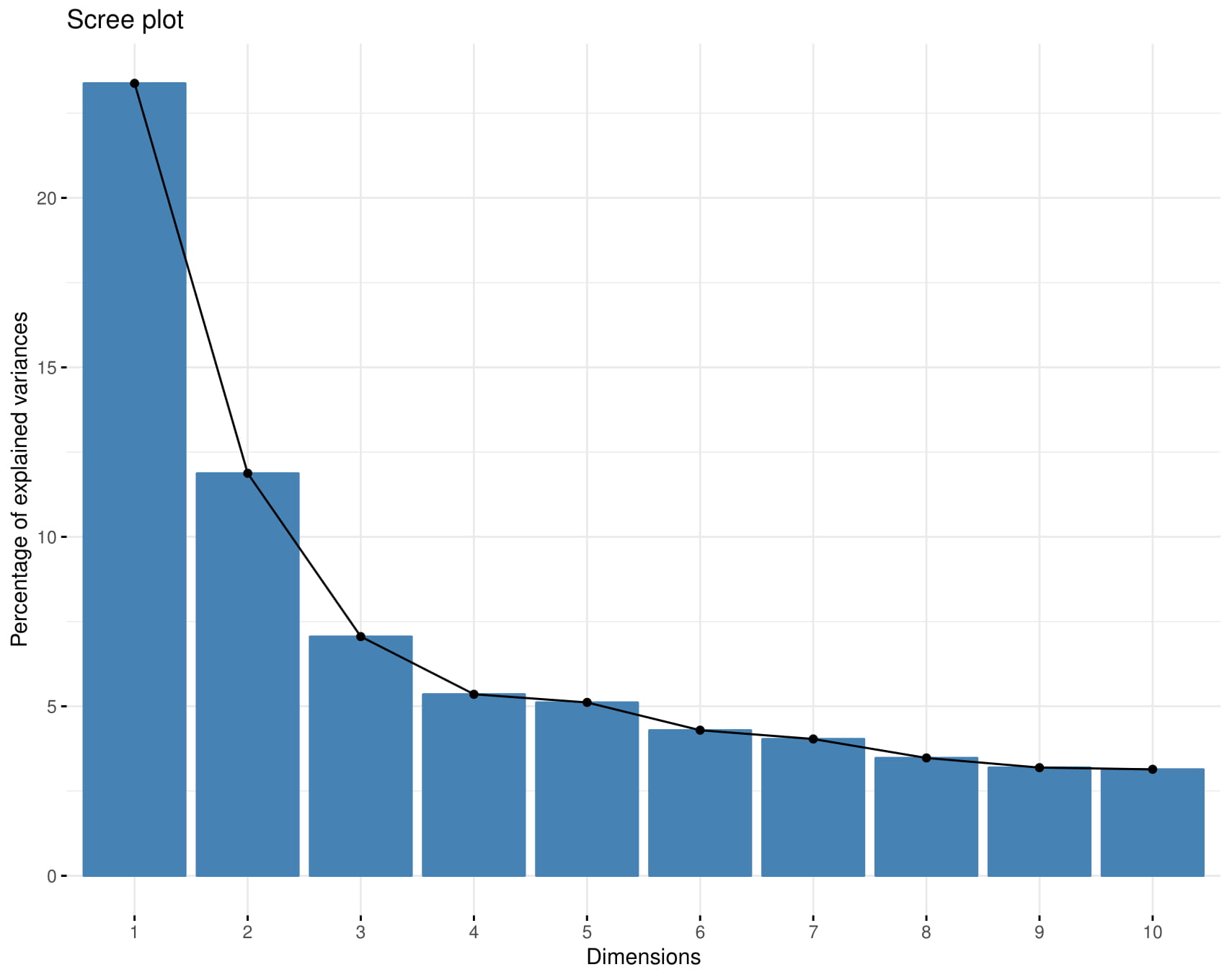


**Figure S3. Plot of eigenvalues of the principal components.**
